# Brain Infection by Group B Streptococcus Induces Inflammation and Affects Neurogenesis in the Adult Mouse Hippocampus

**DOI:** 10.3390/cells12121570

**Published:** 2023-06-06

**Authors:** Katerina Segklia, Rebecca Matsas, Florentia Papastefanaki

**Affiliations:** Laboratory of Cellular and Molecular Neurobiology-Stem Cells, Neurobiology Department, Hellenic Pasteur Institute, 11521 Athens, Greece; rmatsa@pasteur.gr

**Keywords:** group B streptococcus, adult mouse neurogenesis, hippocampus, proliferation, progenitor cells

## Abstract

Central nervous system infections caused by pathogens crossing the blood–brain barrier are extremely damaging and trigger cellular alterations and neuroinflammation. Bacterial brain infection, in particular, is a major cause of hippocampal neuronal degeneration. Hippocampal neurogenesis, a continuous multistep process occurring throughout life in the adult brain, could compensate for such neuronal loss. However, the high rates of cognitive and other sequelae from bacterial meningitis/encephalitis suggest that endogenous repair mechanisms might be severely affected. In the current study, we used Group B Streptococcus (GBS) strain NEM316, to establish an adult mouse model of brain infection and determine its impact on adult neurogenesis. Experimental encephalitis elicited neurological deficits and death, induced inflammation, and affected neurogenesis in the dentate gyrus of the adult hippocampus by suppressing the proliferation of progenitor cells and the generation of newborn neurons. These effects were specifically associated with hippocampal neurogenesis while subventricular zone neurogenesis was not affected. Overall, our data provide new insights regarding the effect of GBS infection on adult brain neurogenesis.

## 1. Introduction

A number of pathogens can enter the brain, elicit compromised cell functions, and induce cell death [1]. Central Nervous System (CNS) infections may be fatal or lead to hugely debilitating outcomes. For example, the consequences of meningitis in surviving infants and adults range from major motor deficits and memory loss to the longer-term development of depression or mood swings [2]. Upon bacterial meningitis/encephalitis, intense inflammatory reaction is observed in the meninges, as well as in the brain parenchyma [3]. Under these conditions, there is extensive brain damage with persisting functional consequences. Vasculitis, focal necrosis in the cortex, damage in the inner ear, and apoptosis in the hippocampus have been reported in patients who died from bacterial meningitis caused by *Streptococcus pneumoniae* [4,5,6]. To cause such damage, infectious agents must cross the blood–brain barrier (BBB), the main factor conferring brain resilience to infection, and invade the brain to interact with different cell populations, including neural stem and progenitor cells (collectively abbreviated herein as NSCs) and their cellular microenvironment in the neurogenic niche [7,8].

Various studies demonstrate that bacterial infections may alter neurogenesis during embryonic development causing neurological disabilities [7], but also during the neonatal period [8,9], as well as in adulthood [10,11,12]. Adult neurogenesis refers to the generation of new neurons from NSCs in the postnatal mammalian brain and is constitutively active throughout life, contributing to brain plasticity. It is mainly restricted in two specific neurogenic regions, particularly the subventricular zone (SVZ) in the lateral walls of the lateral ventricles and the subgranular zone (SGZ) in the dentate gyrus of the hippocampus [13,14]. In rodents, neuroblasts generated from NSCs in the SVZ migrate through the rostral migratory stream to the olfactory bulb where they differentiate into interneurons [13]. On the other hand, neuroblasts generated from NSCs in the SGZ are incorporated in the granule cell layer of the hippocampus where they differentiate to mature granule neurons [14]. Unlike in rodents, in which the major adult neurogenic region resides in the SVZ and is linked to olfaction, in humans the SGZ of the dentate gyrus represents the major neurogenic area and has been associated with cognition [15].

Adult hippocampal neurogenesis is a multistep process that can be followed by the sequential expression of characteristic molecular markers. Radial glia-like NSCs residing in the SGZ and extending their processes through the granule cell layer into the molecular layer of the dentate gyrus are slowly proliferating cells that express the transcription factor Sox2. They give rise to fast-proliferating transit-amplifying progenitors characterized by the expression of the transcription factor achaete-scute homolog-1 (ASCL1, also known as MASH1 from mammalian achaete scute homolog-1), which in turn generate neuroblasts and immature neurons marked by the expression of the microtubule-associated protein doublecortin (DCX). Finally, these differentiate into mature dentate granule neurons that become functionally integrated into existing neuronal networks. Adult neurogenesis offers particular functionality to the human hippocampus as it is related to cognitive functions, such as learning, memory, and certain behaviors [14,16,17]. Interestingly, the impairment of adult hippocampal neurogenesis is linked to cognitive decline in aging, major depression, and Alzheimer’s disease [18,19,20,21,22].

Among the most important bacteria causing meningitis, *Streptococcus agalactiae* (also known as group B streptococcus or GBS) is a commensal bacterium, and a part of the human microbiota colonizing the gastrointestinal and genitourinary tract of up to 30% of healthy human adults, including pregnant women (asymptomatic carriers) [23]. GBS is an opportunistic bacterium and a crucial pathogen for both infants and adults with growing incidence of severe invasive infections [24]. Life-threatening GBS infections are increasingly recognized in the elderly and in individuals with underlying diseases such as diabetes, cirrhosis, and cancer [25,26,27,28,29]. The most common syndrome caused by invasive GBS is bacteremia, but other serious conditions are also attributed to GBS, such as skin and soft tissue infections, diabetes mellitus, endocarditis, osteomyelitis, pneumonia, septicemia, and meningitis with significant cognitive or neurological sequelae [24,30,31,32,33]. Particularly for GBS meningitis—which results in high mortality rates—there are studies reporting that survivors show severe cerebrovascular conditions, such as ischemic stroke and thrombosis [34,35], and experience significant chronic neurologic consequences, such as seizures, cognitive impairment, blindness, hearing loss, hemorrhagic infarction, and hydrocephaly [32,36,37,38,39,40].

GBS strain NEM316 is a serotype III strain that belongs to clonal complex 23 (CC23). Serotype II GBS is responsible for approximately 37% of early-onset and 67% of late-onset neonatal GBS sepsis [41,42], whereas strain BM110 of serotype III that belongs to the hypervirulent complex CC17 is the predominant strain that causes 80% of late-onset meningitis [7]. Previous studies from our group and others have used NEM316 and other GBS strains, both in neonatal and adult mice, to establish infection and examine bacterial meningitis, inflammatory response, and bacterial interactions with the BBB [43,44,45,46,47,48]. However, unlike *Streptococcus pneumoniae-*caused meningitis, the effects of which on neurogenesis have been studied to some extent [11], the consequences of GBS infection on adult neurogenesis have not been addressed, despite reported links to cognitive impairment and other neurological conditions [49,50,51,52,53]. To tackle this question and gain a better understanding of the consequences of GBS infection on adult neurogenesis, in this study we used strain NEM316 of *Streptococcus agalactiae* to establish a mouse model of brain infection and assess the impact of infection on the two major neurogenic niches of the mammalian brain, the hippocampus, and the SVZ at the cellular level.

## 2. Materials and Methods

### 2.1. Ethics Statement

All animal experiments in this study were carried out in the Department of Animal Models for Biomedical Research of the Hellenic Pasteur Institute, under specific pathogen free conditions (Animal House Licenses: EL25BIO011, EL25BIO012, EL25BIO013), in strict compliance with the European and National Laws for Laboratory Animals Use (Directive 2010/63/EU and Presidential Decree 56/2013), with FELASA recommendations for euthanasia and the Guide for the Care and Use of Laboratory Animals of the National Institutes of Health. All animal work was conducted according to protocols approved by the Institutional Animal Protocols Evaluation Committee. License No 6317/27-11-2017 for experimentation was issued by the Greek authorities, i.e.,the Veterinary Department of Attiki Prefecture. The preparation of this manuscript was done in compliance with ARRIVE (Animal Research: Reporting of In Vivo Experiments) guidelines.

### 2.2. Mouse Infection

To examine the effects of GBS brain infection on neurogenesis, 8 to 10 week-old male CD-1 mice (body weight, 40.99 ± 3.62 g [mean ± standard deviation]) were randomly grouped and injected intravenously (i.v.) via the tail vein, either with 10^8^ cfu of GBS (NEM316 strain) suspension (injection volume, 100 μL) or with an equal volume of saline (control mice) (n = 30 mice for the GBS group and n = 13 for the saline group). For euthanasia, mice were deeply anaesthetized by intraperitoneal (i.p.) injection of a mixture containing ketamine (Imalgene 1000, MERIAL, Lyon, France; 100 mg/kg of body weight) and xylazine (Rompun, Bayer, Leverkusen, Germany; 10 mg/kg of body weight) followed by transcardial paraformaldehyde perfusion, at 72 h post infection. Two hours before the end point, 5-bromo-2-deoxyuridine (BrdU) (50 mg/kg of body weight) was injected intraperitoneally (i.p.) to label proliferating cells going through the S-phase of the cell cycle. Following dissection, brains were processed for cryostat sectioning, immunofluorescence, confocal microscopy, and image analysis.

### 2.3. Immunohistochemistry

After transcardial perfusion with 4% paraformaldehyde in phosphate-buffered saline (PBS), the brains of infected and control mice were dissected out, post-fixed in the same fixative, cryoprotected in 30% *w*/*v* sucrose solution in PBS for 2 days at 4 °C, embedded in O.C.T. compound (VWR Chemicals, Monroeville, PA, USA), and frozen at −80 °C. Series of coronal 20 μm-thick sections were collected on Superfrost Plus microscope slides and stored at −20 °C until further processing. The cryosections were thawed and subjected to antigen retrieval in 10 mM sodium citrate solution, pH 6.0, followed by 1 h blocking of non-specific sites with 5% *v*/*v* normal donkey serum (NDS) in PBS, with simultaneous permeabilization using 0.1% *v*/*v* Triton X-100. Primary antibodies diluted in 2.5% NDS in PBS were applied overnight at 4 °C, followed by incubation with the appropriate secondary antibodies for 2 h at room temperature. The following primary antibodies were used: mouse anti-Ki67 (1:1000; Beckton Dickinson, Franklin Lakes, NJ, USA), rat anti-BrdU (1:300; Bio-Rad Antibodies, Oxford, UK), rabbit anti-Sox2 (1:1000; Abcam, Cambridge, UK), mouse anti-achaete-scute homolog 1 (MASH1; 1:100; Beckton Dickinson, Franklin Lakes, NJ, USA), goat anti-doublecortin (DCX; 1:100; Santa Cruz Biotechnology, Dallas, TX, USA), rabbit anti-ionized calcium-binding adapter molecule 1 (Iba1; 1:300; WAKO, FUJIFILM Wako Pure Chemical Corporation, Osaka, Japan), rat anti-cluster of differentiation 68 (CD68; 1:150; Bio-Rad Antibodies, Oxford, UK), mouse anti-GFAP (1:500; Merck, Rahway, NJ, USA), rat anti-CD3 (1:100; R&D Systems, Minneapolis. MI, USA), rabbit anti-CD45 (1:100; Cell Signaling Technology, Danvers, MA, USA), rabbit anti-Caspase-3 (1:400; Cell Signaling Technology, Danvers, MA, USA), rabbit anti-GBS [gift from Dr. S. Dramsi, 1:300; [48], rabbit anti-brain derived neurotrophic factor (BDNF; 1:100; Santa Cruz Biotechnology, Dallas, TX, USA), mouse anti-vesicular glutamate transporter 1 (vGlut1; 1:500; Merck-Millipore, Danvers, MA, USA), and rabbit anti-Parvalbumin (1:100; Invitrogen, Waltham, MA, USA). Secondary antibodies (all from Thermo Fisher Scientific, Waltham, MA, USA) used for immunofluorescence were conjugated with Alexa Fluor 488, 546, and 647 and cell nuclei were counterstained with 4′,6-diamidino-2-phenylindole (DAPI; 1:1000; Thermo Fisher Scientific, Waltham, MA, USA). Prolong Gold antifade curing mountant (Cell Signaling Technology, Danvers, MA, USA) was used for mounting. Images were acquired using Leica TCS SP8 confocal microscope.

### 2.4. Image Analysis

*Cell counts in the dentate gyrus and the subventricular zone.* For each animal, confocal images from 3 brain sections (SVZ coronal coordinates: Bregma 0.85 to −0.11 mm; DG coronal coordinates: Bregma −1.43 to −2.27 mm) were acquired and the number of cells positive for each specific marker was counted using ImageJ (NIH, USA) [54] in the regions of interest (ROI), “blindly” as to the group of animals. The threshold was set at a constant value for each marker. Counts were averaged from 3 sections per ROI per animal. Adobe Photoshop was used to assemble the figures.

*Fluorescence Intensity.* For evaluation of BDNF expression in the hippocampus, mean grey value (pixels) was measured. Briefly, single channel stacks of confocal images were acquired under the same settings (constant gain and offset values, 3× averaging, 1024 × 1024 resolution, 1-μm step size). Quantification of mean grey value was performed using ImageJ software by a blind observer, after free-hand selection of the ROI and setting the threshold at a constant value. Measurements on each single image of the confocal stack were added up and normalized to the area of the ROI. Three animals were analyzed per group and the values from the three sections were averaged per mouse.

### 2.5. Statistical Analysis

Statistical analysis of the data and preparation of graphs were performed in GraphPad Prism software v. 7 (GraphPad, La Jolla, CA, USA). An unpaired two-tailed Student’s *t*-test was used to compare values between groups (values followed a normal distribution according to Kolmogorov–Smirnov normality test). Comparison of survival curves was performed using the Log-rank test. *p*-values lower than 0.05 were considered significant.

## 3. Results

### 3.1. GBS Infection Elicits Neurological Deficits and Causes Death in Mice

**Figure 1:**
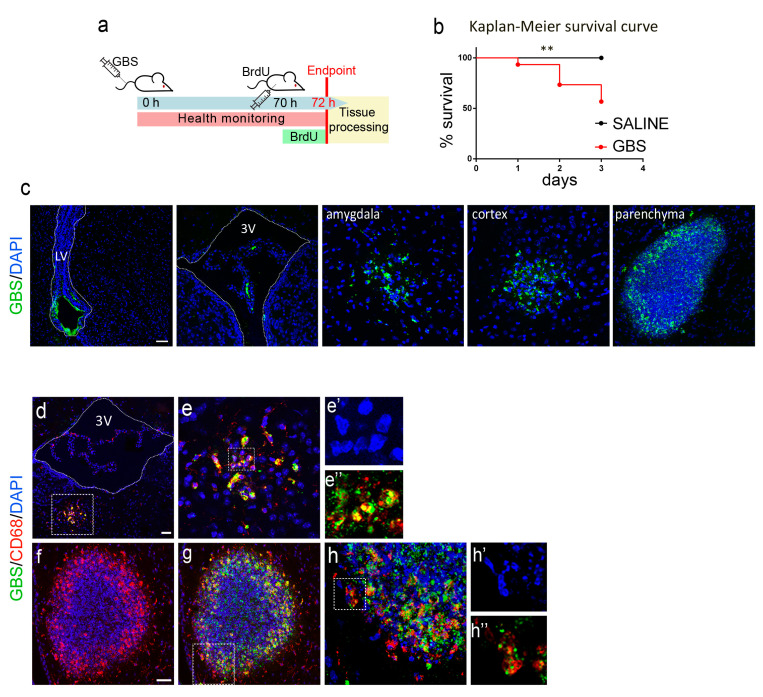
Experimental design and hallmarks of the GBS infection mouse model. (**a**) Schematic representation of the experimental outline followed for GBS intravenous (i.v.) infection in adult mice, health monitoring, and tissue processing at 72 h. (**b**) Kaplan–Meier survival curves of mice intravenously injected with GBS (n = 30) or saline (n = 13). Log-Rank test *p* = 0.0066 (**: *p* ≤ 0.001). (**c**) Confocal images of sections from the indicated brain regions of infected mice immunostained for GBS (green). (**d**–**h″**) Confocal images of brain sections from infected mice immunostained for GBS (green) and CD68 (red), at 72 h after i.v. injection of GBS. Nuclei are counterstained with DAPI (blue). Scale bars (c), 50 μm; (**d**,**f**), 40 μm. The dotted rectangles in d are shown at higher magnification in adjacent micrographs as well as those in g and h. BrdU, 5-bromo-2-deoxyuridine; GBS, Group B Streptococcus; LV, lateral ventricle; 3V, third ventricle; DAPI, 4′,6-diamidino-2-phenylindole; CD68, Cluster of Differentiation 68.

A group of mice (n = 30) were i.v. injected with 10^8^ cfu of the GBS strain NEM316 to be euthanized at 72 h for the immunohistochemical analysis of adult neurogenesis (Figure 1a). Mice in the GBS group presented with morbidity indicative of systemic infection and with neurological deficits, such as unilateral palsy, immobilization, and imbalance, as well as other mood aberrations, including isolation and a lack of explorative behavior, as previously described [48]. The survival curve of the GBS-infected group, compared with that of the saline-injected group (n = 13) is illustrated in Figure 1b. Only 57% of the original population in the GBS group survived by 72 h.

### 3.2. GBS Infection Induces Inflammation and Peripheral Immune Cell Infiltration in the Mouse Brain

We validated the presence of GBS in the brains of surviving mice, sacrificed at 72 h after infection (Figure 1a). Using immunofluorescence, we detected GBS in the choroid plexuses and around the lateral and third ventricles, in the amygdala and cortex as well as in clusters sporadically found in the brain parenchyma (Figure 1c). We then asked if the presence of GBS elicited an inflammatory immune response within the brain. Double immunofluorescence for GBS and CD68, a marker for activated macrophage/microglial cells, revealed the co-localization of GBS staining in CD68^+^ cells, indicating an ongoing process of microglial/macrophage activation and phagocytosis (Figure 1d,e″). This was particularly evident in the case of clustered cells in the parenchyma (Figure 1f–h″), where CD68 was immunodetected in close proximity with or surrounding GFP, apparently reflecting activated CD68^+^ microglia/macrophages engulfing GFP^+^ bacteria for phagocytosis.

**Figure 2:**
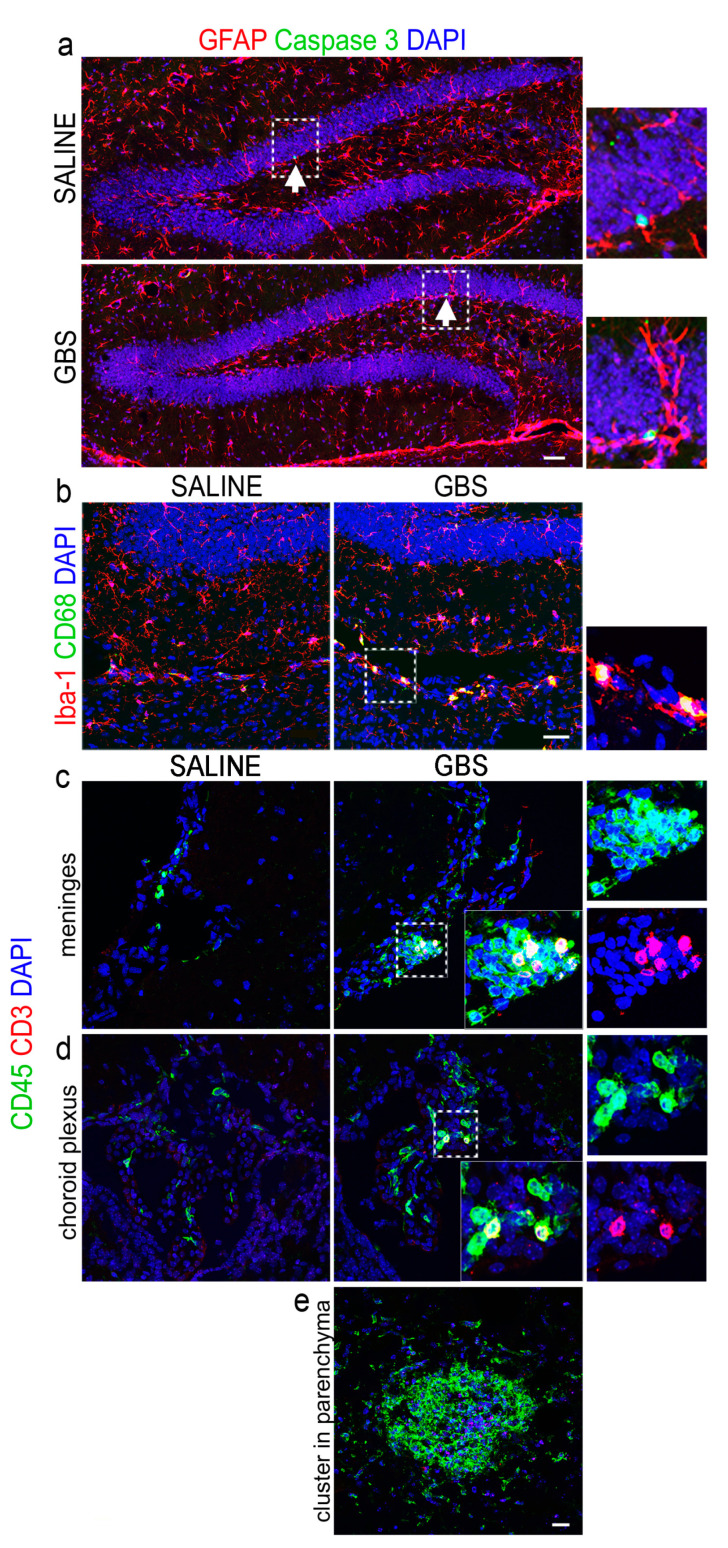
GBS brain infection and neuroinflammation. Confocal images of coronal brain sections from control (saline-treated) or GBS-infected mice (as indicated) at the hippocampal dentate gyrus, double-labeled for GFAP (red) and Caspase-3 (green) (**a**) or Iba-1 (red) and CD68 (green) (**b**). Nuclei are counterstained with DAPI (blue). The arrows in (**a**) point to Caspase-3^+^ cells while the dotted rectangles in (**a**,**b**) are shown at higher magnification. (**c**,**d**) are confocal images from meninges and the choroid plexus, respectively, double-labeled for CD3 (red) and CD45 (green), at 72 h after i.v. injection of saline (control) or GBS. Nuclei are counterstained with DAPI (blue). The dotted rectangles are also shown at higher magnification. (**e**) Micrograph illustrating a GBS cluster (green) in brain parenchyma. Scale bars (**a**), 100 μm; (**b**), 40 μm; (**e**), 20 μm. GBS, Group B Streptococcus; GFAP, glial fibrillary acidic protein; Iba-1, Ionized calcium binding adaptor molecule 1; CD68, Cluster of Differentiation 68; DAPI, 4′,6-diamidino-2-phenylindol.

Given the initial observation of active inflammation in the brains of infected mice, we further examined the two major features of neuroinflammation in the hippocampal dentate gyrus, namely astrogliosis, estimated by the levels of glial fibrillary acidic protein (GFAP) expression in astrocytes, and microgliosis, assessed by double immunostaining for ionized calcium binding adaptor molecule 1 (Iba-1) and CD68 that, when co-expressed, label-activated macrophage/microglial cells. The immunodetection of GFAP did not present apparent changes in the GBS-injected group as compared with the saline group, indicating an absence of astrogliosis (Figure 2a). On the other hand, double labeling for Iba-1 and CD68 revealed the presence of double positive cells (activated microglia), particularly in brain areas adjacent to the hippocampus of the GBS-infected mice, as shown in Figure 2b. The examination of apoptosis by immunostaining for cleaved Caspase-3 in the dentate gyrus of the infected and control groups did not show any measurable differences, at 72 h after GBS infection (Figure 2a).

Further, we assessed the infiltration of peripheral T and B lymphocytes. Double immunostaining for CD3 and CD45, which mark T (CD3^+^/CD45^+^) and B (CD3^−^/CD45^+^) lymphocytes, respectively, revealed the infiltration of both T and B cells in the brain of GBS-infected mice, particularly in areas such as the meninges and the choroid plexuses (Figure 2c,d). Further, we observed the presence of T and B cellular clusters in the brain parenchyma of infected animals that were not seen in the control group (Figure 2e).

### 3.3. GBS Brain Infection Affects Adult Hippocampal Neurogenesis in the SGZ of the Dentate Gyrus

**Figure 3:**
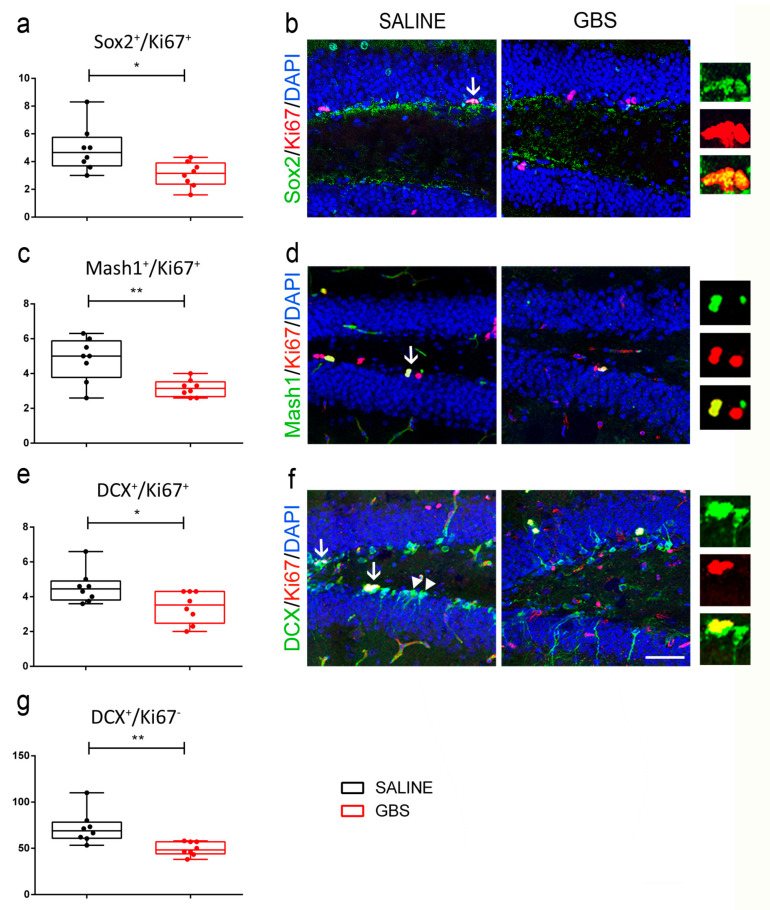
Effect of GBS brain infection on the neurogenic niche of the subgranular zone in the dentate gyrus of the hippocampus. Quantification and representative confocal images of proliferating neural stem/progenitor cells (Sox2^+^/Ki67^+^, (**a**,**b**)), transit amplifying cells (MASH1^+^/Ki67^+^, (**c**,**d**)), proliferating neuroblasts (DCX^+^/Ki67^+^, (**e**,**f**); indicated by arrows), and newborn neurons (DCX^+^/Ki67^−^, (**g**,**f**); indicated by arrowheads) in the subgranular zone of the hippocampus of GBS- or saline-injected mice. Nuclei are counterstained with DAPI (blue). Box plots with whiskers illustrate all data points, mean, min and max values. Scale bar, 50 μm. Student’s t-test two tailed values: *: *p* ≤ 0.01, **: *p* ≤ 0.001. GBS, Group B Streptococcus; DCX, doublecortin; Sox2, SRY (sex determining region Y)-box 2; MASH1, mammalian achaete-scute homolog 1; DAPI, 4′,6-diamidino-2-phenylindole.

To analyze the response of the hippocampal neurogenic niche in the SGZ of the dentate gyrus to GBS infection, we performed multiple immunofluorescence labeling on coronal brain sections, at 72 h post infection. We assessed the proliferation of NSCs using the proliferation marker Ki67 and markers specific to the different cell stages of neurogenesis. The total number of proliferating cells estimated by Ki67 showed a decreasing trend in GBS-infected mice as compared with the saline group, which did not reach statistical significance (not shown). Nevertheless, when we looked into the different proliferating cell populations present in the SGZ neurogenic niche we identified significant differences between the two groups of mice. In particular, Sox2^+^/Ki67^+^ proliferating NSCs were significantly reduced upon GBS infection (number of cells per section; 4.9 ± 0.58 in saline group vs. 3.08 ± 0.31 in GBS group; n = 8 mice per group; *p* = 0.016; Figure 3a,b). In addition, MASH1^+^/Ki67^+^ cells, corresponding to the fast-proliferating transit-amplifying population of precursor cells, were significantly decreased in GBS-infected mice (number of cells per section; MASH1^+^/Ki67^+^: 5 ± 1.47 in the saline group vs. 3.2 ± 0.26 in the GBS group; n = 8 mice per group; *p* = 0.006; Figure 3c,d). Similarly, proliferating neuroblasts (DCX^+^/Ki67^+^) were significantly reduced in the GBS-infected mice, as compared with the control saline-injected mice (number of cells per section; 4.55 ± 0.33 in saline group vs. 3.4 ± 0.32 in GBS group; n = 8 mice per group; *p* = 0.027; Figure 3e,f). In line, there was a significant reduction in the number of post-mitotic newborn neurons (DCX^+^/Ki67^−^) in the SGZ of the GBS-infected mice (number of cells per section; 72.13 ± 6.15 in saline vs. 49.53 ± 2.58 in GBS group; n = 8 mice per group; *p* = 0.0044; Figure 3g,f).

**Figure 4:**
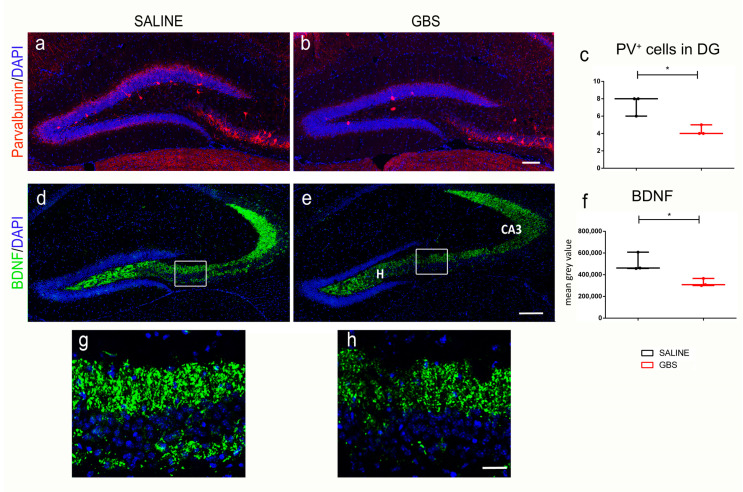
Reduced immunodetection of Parvalbumin^+^ neurons and BDNF in the hippocampus after GBS infection. Confocal images of Parvalbumin-expressing cells in the hippocampus of saline-treated or GBS-injected mice (**a**,**b**), and quantification of Parvalbumin^+^ cells in the dentate gyrus (**c**). Confocal images and quantification of BDNF immunofluorescence in the hippocampal area of saline-treated or GBS-injected mice (**d**–**f**). (**g**,**h**) Higher magnification of boxed areas in (**d**,**e**), respectively. Nuclei are counterstained with DAPI (blue). BDNF clearly marks the mossy fibre pathway. Box plots with whiskers illustrate all data points, mean, min and max values. Scale bars (**b**,**e**), 100 μm; (**h**), 20 μm. H, Hilus; CA, Cornu Ammonis; BDNF, brain-derived neurotrophic factor; DAPI, 4′,6-diamidino-2-phenylindole. Student’s *t*-test two tailed values: *: *p* ≤ 0.01.

Further, we examined possible changes in the population of parvalbumin-expressing interneurons in the hippocampal neurogenic niche, given their known roles in supporting the survival of newborn neurons in this area [55,56]. By immunofluorescence, we observed a significant decrease in the numbers of parvalbumin^+^ cells in the dentate gyrus (number of cells per section; 7.33 ± 0.67 in saline mice vs. 4.33 ± 0.33 in GBS mice; n = 3 mice per group; *p* = 0.0158; Figure 4a–c).

Finally, we investigated the correlation between hippocampal damage, which is known to contribute to impaired learning and memory, and the brain-derived neurotrophic factor (BDNF), the most widespread growth factor in the brain [57]. The BDNF is primarily expressed in hippocampal mossy fiber projections, i.e., the unmyelinated axons of granule cells in the dentate gyrus that terminate on mossy cells and in CA3 pyramidal neurons that are important for spatial memory formation and consolidation [58,59]. Importantly, the BDNF regulates neuronal survival, synaptic transmission, and synaptic plasticity [60]. Immunofluorescence labeling for the BDNF showed a statistically significant decrease in the hilus and the CA3 hippocampal region, at 72 h after GBS infection (mean gray value; 50.89 ± 4.924 in saline mice vs. 32.49 ± 2.058 in GBS mice; n = 3 mice per group; *p* = 0.0261; Figure 4d–f).

### 3.4. Adult SVZ Neurogenesis Is Not Affected upon GBS Infection

**Figure 5:**
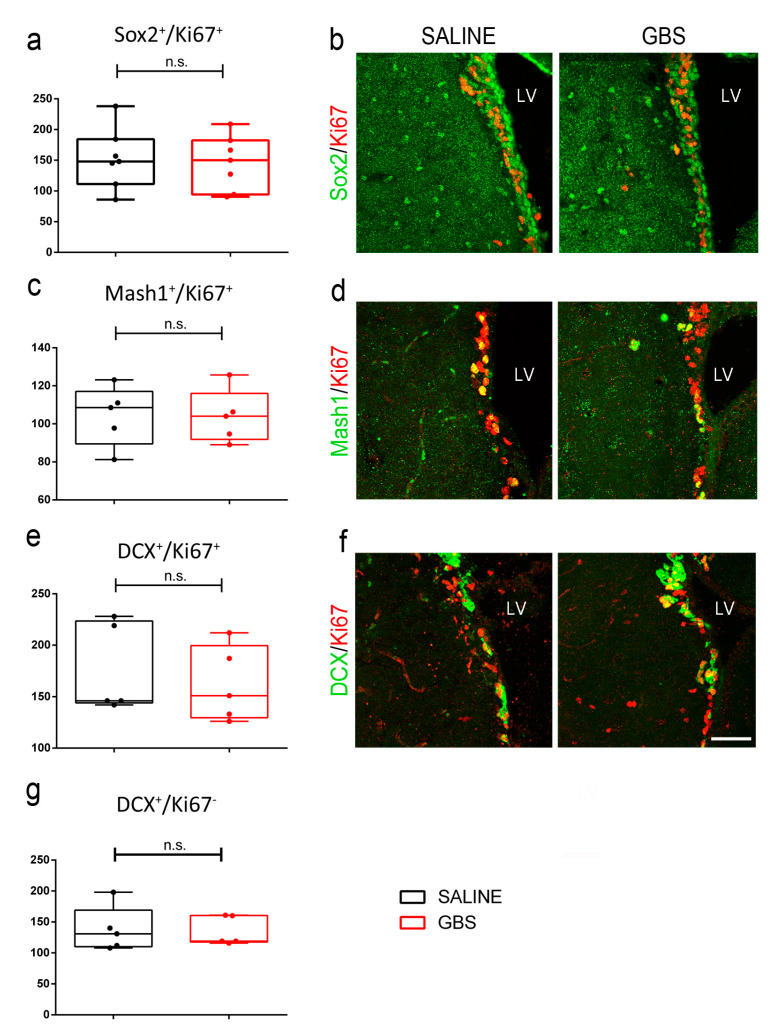
Effect of GBS brain infection on the neurogenic niche of the subventricular zone. Quantification and representative confocal images of proliferating neural stem/progenitor cells (Sox2^+^/Ki67^+^, (**a**,**b**)) and transit amplifying cells (MASH1^+^/Ki67^+^, (**c**,**d**)), proliferating neuroblasts (DCX^+^/Ki67^+^, (**e**,**f**)), and newborn neurons (DCX^+^/Ki67^−^, (**g**,**f**)) in the subventricular zone of GBS- or saline-injected mice. Box plots with whiskers illustrate all data points, mean, min and max values. Scale bar, 50 μm. Student’s *t*-test two tailed values: n.s.: *p* > 0.05. LV, lateral ventricle; GBS, Group B Streptococcus; DCX, doublecortin; Sox2, SRY (sex determining region Y)-box 2; MASH1, mammalian achaete-scute homolog 1; DAPI, 4′,6-diamidino-2-phenylindole.

Given the effect of GBS infection on the neurogenic population of the SGZ, we asked if there is a similar response in the neurogenic niche of the SVZ. However, unlike the SGZ, in the SVZ, no significant differences were noted between the two groups in any of the proliferating precursor cell types examined (number of cells per section; Sox2^+^/Ki67^+^ proliferating NSCs: 152.8 ± 18.6 in saline vs. 145.7 ± 16.75 in the GBS group, n = 7 mice per group, *p* = 0.8; Figure 5a,b; Mash1^+^/Ki67^+^ fast proliferating transit amplifying precursor cells: 104.4 ± 7.05 in saline vs. 104 ± 6.3 in the GBS group, n = 5 mice per group, *p* = 0.96; Figure 5c,d; DCX^+^/Ki67^+^ proliferating neuroblasts: 176.2 ± 19.3 in saline vs. 161.8 ± 16.41 in the GBS group, n = 5 mice per group, *p* = 0.58; Figure 5e,f). In agreement, the post-mitotic DCX^+^/Ki67^−^ newborn neurons were not different in numbers between the two groups (number of cells per section; 137.8 ± 16.1 in saline mice vs. 135 ± 10.4 in GBS mice; n = 5 mice per group; *p* = 0.88; Figure 5g,f). Additionally, no significant differences between the various populations of SVZ cells were noted between the two groups of mice, when we monitored a 2 h cohort of cells progressing through the S-phase of the cell cycle (BrdU^+^) (Supplementary Figure S1).

## 4. Discussion

Central nervous system (CNS) infections are extremely harmful and can result in fatal outcomes or long-term neurological disabilities in surviving infants and adults, including cognitive deficits. *Streptococcus agalactiae* or GBS is among the most important pathogen causing human meningitis with detrimental consequences in infected subjects. For bacterial meningitis to develop, the dissemination of the pathogen from blood circulation to the meningeal walls is necessary. To invade the brain, GBS must first interact with and penetrate the BBB. We have previously shown that the GBS surface lipoprotein B leucine-rich (Blr) is essential for BBB crossing and pathogenicity, enabling GBS to invade the mouse brain, whereas its interaction with the host (V)LDL receptor LpR2 expressed in the BBB was demonstrated in Drosophila [48]. Several other virulence factors have also been described, such as the GBS surface antigen I/II protein BspC that promotes pathogenicity through interaction with the intermediate filament protein Vimentin [61].

Given the severe neurological defects that have been attributed to pneumococcal meningitis, here we sought to investigate the effects of GBS infection on adult neurogenesis, which is involved in memory and learning, whilst disrupted neurogenesis in the hippocampus is implicated in cognitive impairment and mood disorders [51,62,63,64]. Our data demonstrate that GBS infection compromises hippocampal neurogenesis, whereas it has no detectable effect on SVZ neurogenesis. Since the SVZ is the richest source of NSCs in mice and the largest neurogenic niche in this species, it is possible that small differences may not be detectable, or may be counterbalanced by compensatory host responses.

Our observations on the decline of hippocampal neurogenesis are relevant, highlighting potential consequences of GBS infection in humans. Given that, at the time of the analysis, bacteria were not detectable in the neurogenic region of the dentate gyrus, it is likely that the effects elicited by GBS are mediated via the increased inflammatory response observed. Previous studies have shown that inflammation, for example that induced by lipopolysaccharide, which is a major component of a gram-negative bacteria membrane, results in the activation of microglia close to the region where new neurons are produced. The activation of the brain’s resident immune cells in turn leads to the impairment of hippocampal neurogenesis [65,66,67,68]. Indeed, there appears to be a significant inverse correlation between the number of surviving new hippocampal neurons and the number of activated microglia [69,70,71]. In addition, various studies have described neuroinflammation associated with CNS infection [72], autoimmune disease [73], and stroke [74]. In the current study, the presence of CD68^+^ microglia/macrophages comes in agreement with previous reports showing that the activation of microglia is a major feature in an inflamed environment after CNS damage [75,76]. A previous study has also demonstrated that endogenous microglia play an important role in the pathology of the inflamed brain, in a mouse model of bacterial meningitis induced by *Streptococcus pneumoniae* [77]. Moreover, peripheral inflammation, as observed in our case, has been associated with disturbances in hippocampal neurogenesis. In particular, pro-inflammatory cytokines released in the periphery are thought to be involved in peripheral immune system-to-brain communication by activating resident microglia in the brain [78]. Hippocampal damage may be the consequence of a complex process triggered by bacteria and their components and depends on the host reaction, as well as on the phase of the disease. Increased apoptosis specifically in dentate gyrus cells of the hippocampus has been described in the majority of patients that died from bacterial meningitis, but not in control patients that died from rapidly fatal non-neurological diseases [79,80]. The time course of apoptotic cell death in the SGZ of the hippocampus has been assessed in a rat model of bacterial meningitis caused by *Streptococcus pneumoniae* [8]. An increase in active Caspase-3 positive cells was mainly observed during the acute phase of infection (18–24 h), with a peak at 36 h post infection, whereas the number of apoptotic cells returned to control levels in the sub-acute phase at 36–72 h after infection [8]. In line, we did not detect any measurable differences in the numbers of active Caspase-3 positive apoptotic cells, at 72 h post GBS infection. In addition, hippocampal injury seems to be dependent on the type of pathogen that the host is infected with. Interestingly, the apoptotic cells that are affected by *Streptococcus pneumoniae* are immature neurons [8], whereas in agreement with our observations, GBS infection appears to affect the populations of both immature and mature neurons [8,81,82]. We recorded a prominent reduction in the population of parvalbumin interneurons, which are crucial for the survival of newborn granule neurons in the SGZ [55]. Further, a reduction at the levels of the pro-survival and differentiation-promoting neurotrophic factor BDNF was also noted, apparently contributing to the overall deficit in neurogenesis with conceivable functional implications. Notably, previous studies have shown that the functional cognitive deficit, such as an impairment in learning and memory, caused by experimental pneumococcal meningitis in adult rodents, is exacerbated by the selective loss of the BDNF in infected conditional knock-out mice [62,83].

We and others have clearly shown a decrease in hippocampal neurogenesis at 72 h after bacterial infection. Nevertheless, in mouse and rabbit models of intracerebral or intracisternal infection with *S. pneumoniae*, an enhancement in neurogenesis has been suggested in the subgranular layer of the dentate gyrus, particularly at earlier time points post infection, as well as increased levels of BDNF [49,50]. Different infection routes and the use of a different pathogen, as well as the fact that animals were placed under an antibiotic regime as early as 24 h post infection, may account for these observations. Alternatively, or in addition, it is possible to envisage an initial host response to increase neurogenesis in order to counteract the adverse effects of infection and to compensate neuronal death. However, at later stages of infection, it seems that this endogenous brain repair mechanism is not sufficient to alleviate the consequences of noxious stimuli, and neurogenesis eventually declines. Higher concentrations of BDNF, as well as increased neuronal proliferation, have also been reported in the cerebrospinal fluid of children and adult patients with meningitis or encephalitis [84,85,86]. Again, the impact of different pathogens examined the different routes of infection, and the variety in proinflammatory agents acting at the time of investigation need to be considered. Nevertheless, cognitive impairment is a persistent condition in surviving patients, suggesting that the reduction in specific cell types cannot be reversed.

To conclude, our study demonstrates that GBS-related meningitis compromises adult neurogenesis, specifically in the hippocampus, and is associated with reduced numbers of proliferating NSCs and neuroblasts, resulting in reduced numbers of post-mitotic neurons in the SGZ. Additionally, we observed reduced BDNF levels, as well as a decrease in the number of Parvalbumin^+^ interneurons, both of which support adult neurogenesis. These effects could be mediated directly by the interaction of GBS endo- or/and exo-toxins with the host brain cell populations and/or indirectly by the inflammatory response of activated microglia and infiltrating peripheral immune cells. In all, our data provide novel insights into GBS infection and the resulting neurological deficits, broadening our understanding of disease pathogenesis.

## Data Availability

All data are contained within the article.

## Supplementary Materials

The following supporting information can be downloaded at: https://www.mdpi.com/article/10.3390/cells12121570/s1, Figure S1: Effects of GBS brain infection on BrdU-labeled cells in the subventricular zone.
