# Supplementary material for: Brain Infection by Group B Streptococcus Induces Inflammation and Affects Neurogenesis in the Adult Mouse Hippocampus
Source: Cells. 2023 Jun 6;12(12):1570. doi: 10.3390/cells12121570 (PMC10296580; doi:10.3390/cells12121570)
Supplement: Supplementary file 1 [file cells-12-01570-s001.zip › cells-2405804-supplementary.pdf]

cells MDPI

Supplementary

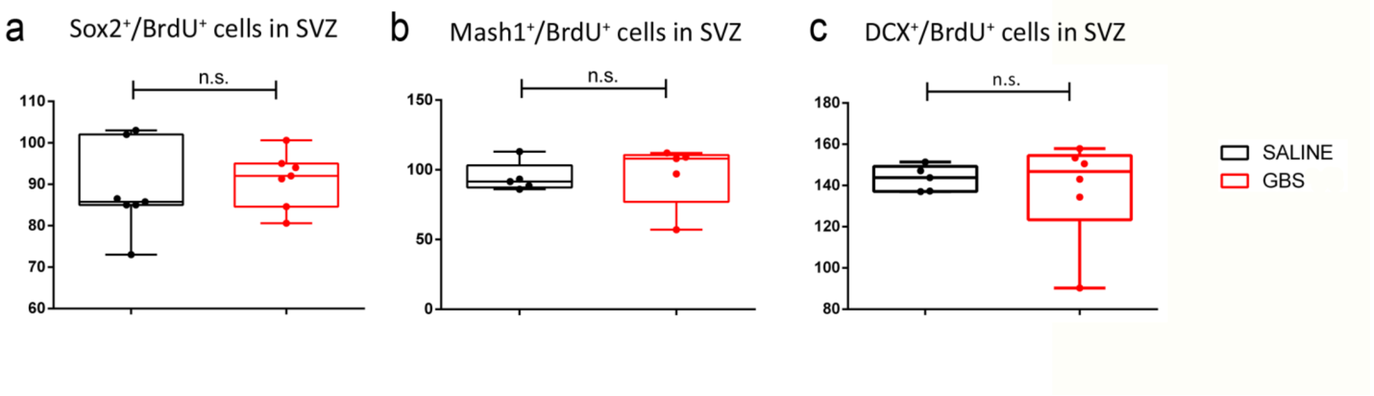

Figure S1. Effect of GBS brain infection on BrdU-labeled cells in the subventricular zone. Quantification of proliferating neural stem/progenitor cells (Sox2⁺/BrdU⁺, **a**), transit amplifying cells (Mash1⁺/BrdU⁺, **b**), and neuroblasts (DCX⁺/BrdU⁺, **c**) in the subventricular zone of GBS or saline-injected mice. Box plots with whiskers illustrate all data points, mean, min and max values. GBS, Group B Streptococcus; Sox2, SRY (sex determining region Y)-box 2; Mash1, mammalian achaete-scute homolog 1 ; DCX, doublecortin; BrdU; SVZ, subventricular zone.
